# Identificationof a novel linear epitope on the porcine reproductive and respiratory syndrome virus nucleocapsid protein, as recognized by a specific monoclonal antibody

**DOI:** 10.3389/fimmu.2023.1165396

**Published:** 2023-04-18

**Authors:** Yuening Cheng, Miaoli Wu, Li Xiao, Mengdi Zhang, Bihong Huang, Feng Cong, Li Yi

**Affiliations:** ^1^ Institute of Special Economic Animal and Plant Science, Chinese Academy of Agricultural Sciences, Changchun, China; ^2^ Guangdong Laboratory Animals Monitoring Institute and Guangdong Provincial Key Laboratory of Laboratory Animals, Guangzhou, China

**Keywords:** PRRSV, N protein, monoclonal antibody, linear B-cell epitope, identification

## Abstract

**Introduction:**

Porcine reproductive and respiratory syndrome virus (PRRSV) remains one of the most threatening pathogens of swine. The nucleocapsid (N) protein is the major structural protein of the virus and has been used as a PRRSV diagnostic antigen due to its high level of inherent immunogenicity.

**Methods:**

The recombinant PRRSV N protein was generated by the prokaryotic expressing system and used to immunized mice. Monoclonal antibodies against PRRSV were produced and validated by western blot analysis and indirect immunofluorescence analysis. In this study, the linear epitope of a specific monoclonal antibody mAb (N06) was subsequently identified by enzyme-linked immunosorbent assays (ELISA) using the synthesized overlapping peptides as antigens.

**Results:**

According to the results of western blot analysis and indirect immunofluorescence analysis, mAb (N06) was capable of recognizing the native form as well as the denatured form of PRRSV N protein. The results of ELISA showed that mAb N06 recognized the epitope NRKKNPEKPHFPLATE, which was consistent with BCPREDS predictions of antigenicity.

**Conclusion:**

All the data suggested that the mAb (N06) can be used as diagnostic reagents for PRRSV detection, while the recognized linear epitope can be useful in epitope-based vaccines development, which is helpful for the control of local PRRSV infections in swine.

## Introduction

Porcine reproductive and respiratory syndrome virus (PRRSV) is a small, positive-sense, single-stranded RNA (ssRNA) virus that causes late-term reproductive failure in pregnant sows, as well as respiratory distress in pigs of all ages (1).The virus was first reported in the USA and is now circulating in most swine-producing countries, causing huge economic losses to the swine industry (2–4). PRRSV vaccines are in widespread use, but outbreaks of porcine reproductive and respiratory syndrome (PRRS) continue to occur in intensive pig production facilities (5, 6). The lack of specific treatments and failure of vaccination has led to poor overall protection against PRRSV, as the protective immune response to PRRSV is poorly understood (7).

PRRSV is a member of the *Arteriviridae* family and can be divided into two genotypes, namely PRRSV-1 subgenus *Eurpobartevirus* and PRRSV-2 subgenus *Ampobartevirus* (8). A high degree of genetic diversity is a significant characteristic of PRRSV. Its genome size is 15.4 kb, and it contains 10 open reading frames (ORFs) that encode 14 non-structural and seven structural proteins (9). The nucleocapsid (N) protein related to immune evasion is highly immunogenic and constitutes up to 40% of the protein content of the virus (10, 11). The early stages of the immune response to PRRSV are primarily directed against the N protein, and the corresponding antibodies can be detected just 1 week after infection, making it an ideal early diagnostic test reagent (12).

The prediction of B- and T-cell epitopes in target antigens is a key step in epitope-driven subunit vaccine design, immunodiagnostic testing, and antibody production (13). Previous studies have demonstrated that the N protein possesses four or five domains of antigenic importance (14). Although these antigenic regions of the N protein are well known, most of the antigenic epitopes have not been precisely identified (15, 16).

In the current study, monoclonal antibodies (mAbs) against PRRSV were obtained by immunizing BALB/c mice with recombinant N protein. The precise B-cell epitopes were further identified when using these mAbs to interact with a series of synthetic peptides that contained N protein segments during indirect enzyme-linked immunosorbent assay (ELISA) analysis. Finally, a novel linear B-cell epitope was confirmed, which will provide valuable information for the diagnosis of PRRSV infection and design of a vaccine against PRRSV.

## Materials and methods

### Virus, plasmids, cells, and peptides

PRRSV strain GDQY (accession no. KY488478.1) was preserved by our laboratory. The cell lines Marc-145 and SP2/0 were maintained in Dulbecco’s modified Eagle medium (DMEM) (Gibco BRL, Paisley, UK), which contained 10% fetal bovine serum (FBS) (Gibco BRL, Paisley, UK). The recombinant plasmid pET28a-N, containing the PRRSV N gene, was synthesized by Sangon Biotech (Shanghai, China). A set of peptides designed according to the N gene was synthesized by GenScript (Nanjing, China) (see below).

### Preparation of recombinant N protein

The recombinant plasmid pET28a-N was transformed into *Escherichia coli* strain BL21 (TransGen Biotech, Beijing, China) for protein expression. The recombinant pET28a-N protein was produced by inducing the positive bacterial strain with 0.1% isopropyl β-D-1-thiogalactopyranoside (IPTG) for 6 hours at 37°C. The harvested cells were lysed before the recombinant protein was purified using Ni-NTA affinity chromatography (GE Healthcare, Chicago, IL, USA) following the manufacturer’s instructions (17). The purified recombinant protein was analyzed with an anti-His-tagged mAb, as well as positive PRRSV serum, by Western blotting (18).

### mAb production

Monoclonal antibodies against the recombinant N protein were generated using hybridoma technology, as previously described (19, 20). Briefly, three female BALB/c mice (aged 6–8 weeks) were injected with 30 μg of purified recombinant N protein, which was emulsified in complete Freund’s adjuvant (Sigma-Aldrich, St. Louis, MO, USA), as the primary immunization. The animals were given three booster injections with the same antigens plus incomplete Freund’s adjuvant at consecutive 2-week intervals. Serum antibody titers were evaluated using an indirect enzyme-linked immunosorbent assay (ELISA) 10 days after the third booster vaccination (21). Splenocytes collected from the mice were fused with SP2/0 cells using polyethylene glycol 1450 (Sigma-Aldrich, St. Louis, MO, USA). Fused cells were then seeded into 96-well plates and selected in DMEM containing hypoxanthine–aminopterin–thymidine (HAT) (Sigma-Aldrich, St. Louis, MO, USA) and 20% FBS (22). The hybridoma cells were maintained in DMEM containing hypoxanthine–thymidine and 20% FBS. Hybridoma supernatants were then analyzed using indirect ELISA (see below). The positive mAb-producing hybridomas were subcloned three times by limiting dilution assay to obtain a single hybrid cell line. Ascites fluid was obtained by intraperitoneal injection of 5 × 10^5^ hybridoma cells into male BALB/c mice treated with 0.5 mL/mouse 2,6,10,14-tetramethylpentadecane (pristane; Sigma-Aldrich, St. Louis, MO, USA). Purified mAbs were prepared from the ascites fluid using HiTrap^®^ Protein G prepacked columns (GE Healthcare, Chicago, IL, USA) as previously described (23).

### Indirect ELISA

For the indirect ELISA analysis, 96-well plates coated with 1 μg/mL recombinant N protein were used to screen samples containing antibodies against the N protein. The samples were collected from immunized mice and hybridoma culture supernatants, as well as antisera fluid. Briefly, the plates were blocked with 5% skim milk (Sigma-Aldrich, St. Louis, MO, USA) for 1 hour at 37°C and washed three times with PBST (1× phosphate-buffered saline containing 0.05% Tween 20) before loading the samples. The plates were then incubated at 37°C for 1 hour and PBST was used to wash away the unconjugated antibodies. Antibody binding was detected by the addition of horseradish peroxidase (HRP)-conjugated goat anti-mouse immunoglobulin G (IgG) (Beyotime, Beijing, China), and absorbance at 630 nm was measured following the addition of the 3,3′5,5′-tetramethylbenzidine (TMB) (Beyotime, Beijing, China) substrate.

### Indirect immunofluorescence

Marc-145 cells were infected with PRRSV GDQY strain at 0.1 multiplicity of infection (MOI), incubated for 1 hour at 37°C, following which the medium was replaced with DMEM containing 2% FBS. At 48 hours post infection, cells were fixed with 4% paraformaldehyde (Sigma-Aldrich, St. Louis, MO, USA) and treated with 0.1% Triton X-100 (Sigma-Aldrich, St. Louis, MO, USA) at room temperature for 10 minutes before they were further blocked with 1% bovine serum albumin (Sigma-Aldrich, St. Louis, MO, USA) at 37°C for 1 hour. Subsequently, the cells were incubated with the mAbs (see above) at 37°C for 1 hour and then washed three times with PBST, followed by incubation with goat anti-mouse IgG H and L chain antibody labeled with fluorescein isothiocyanate (FITC) (Abcam, Cambridge, UK). The cells were visualized using a fluorescent microscope (Leica Microsystems, Wetzlar, Germany) (24).

### Prediction of B-cell epitopes and peptide synthesis

Linear B-cell epitopes of the N protein were predicted using the B-cell epitope prediction server (BCPred: http://ailab-projects1.ist.psu.edu:8080/bcpred/) and BepiPred-3.0 (https://services.healthtech.dtu.dk/service.php?BepiPred-3.0). To improve the accuracy of the prediction, physicochemical properties of amino acid groups were selected instead of single properties. The fixed-length epitope prediction was carried out using the N protein amino acid sequence (accession no. KY488478.1), and the prediction results were further validated with the help of the BepiBlast online server (Immunomedicine Group; BEPIBLAST: B Cell Epitope Prediction using a BLAST-based module). Peptides representing the selected B-cell epitopes were synthesized and purified using high-performance liquid chromatography (HPLC) to > 95% purity (GenScript, Nanjing, China) (**Table 1**). The peptides were tested on mAb conjugation by indirect ELISA analysis (see above).

**Table 1:** Synthesized peptide sequences.

Name	Amino acids sequence and N protein location
N1	^1^MPNNNGKQQKKKKGNGQPVNQLCQM^25^
N2	^20^NQLCQMLGKIIAQQNQSRGKGPGKK^44^
N3	^39^KGPGKKNRKKNPEKPHFPLATEDDV^63^
N4	^58^ATEDDVRHHFTPSERQLCLSSIQTA^82^
N5	^77^SSIQTAFNQGAGTCALSDSGRISYT^101^
N6	^96^GRISYTVEFSLPTQHTVRLIRATASPSA^123^
N31	^39^KGPGKKNRKKNPEKPH^54^
N32	^41^PGKKNRKKNPEKPHFP^56^
N33	^43^KKNRKKNPEKPHFPLA^58^
N34	^45^NRKKNPEKPHFPLATE^60^
N35	^47^KKNPEKPHFPLATEDDV^63^
N341	^45^NRKKNPEKPHF^55^
N342	^46^RKKNPEKPHFP^56^
N343	^47^KKNPEKPHFPL^57^
N344	^48^KNPEKPHFPLA^58^
N345	^49^NPEKPHFPLAT^59^
N346	^50^PEKPHFPLATE^60^

## Results

### Expression of the PRRSV N protein and the generation of anti-N mAb

**Figure 1:**
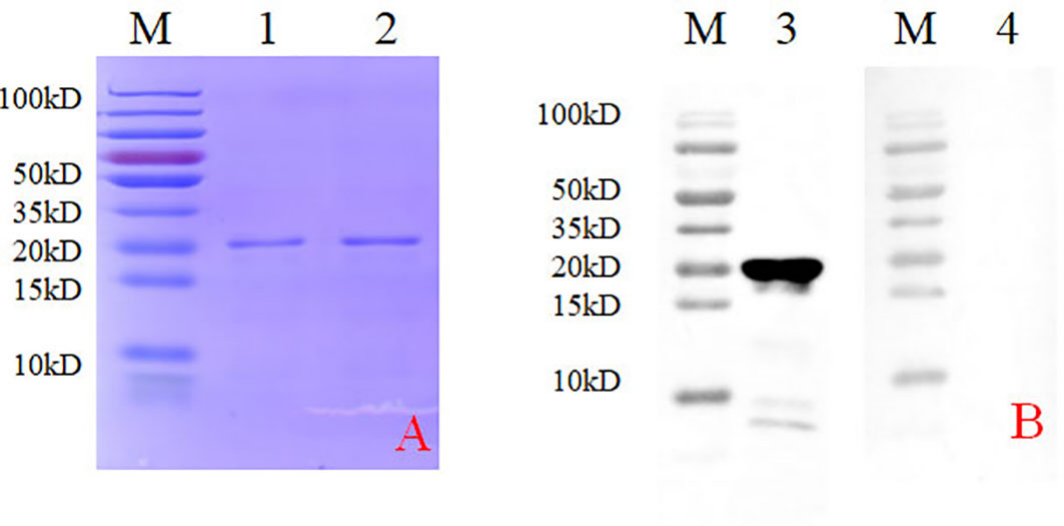
Identification of the recombinant N protein following bacterial expression and Ni-NTA affinity chromatography. **(A)** SDS-PAGE analysis. Lane M: protein marker; lanes 1 and 2: purified recombinant N protein samples. **(B)** Western blotting analysis using anti-His-tagged primary antibody. Lane 3, recombinant N protein; lane 4, negative control; SDS-PAGE, sodium dodecyl sulfate-polyacrylamide gel electrophoresis.

**Figure 2:**
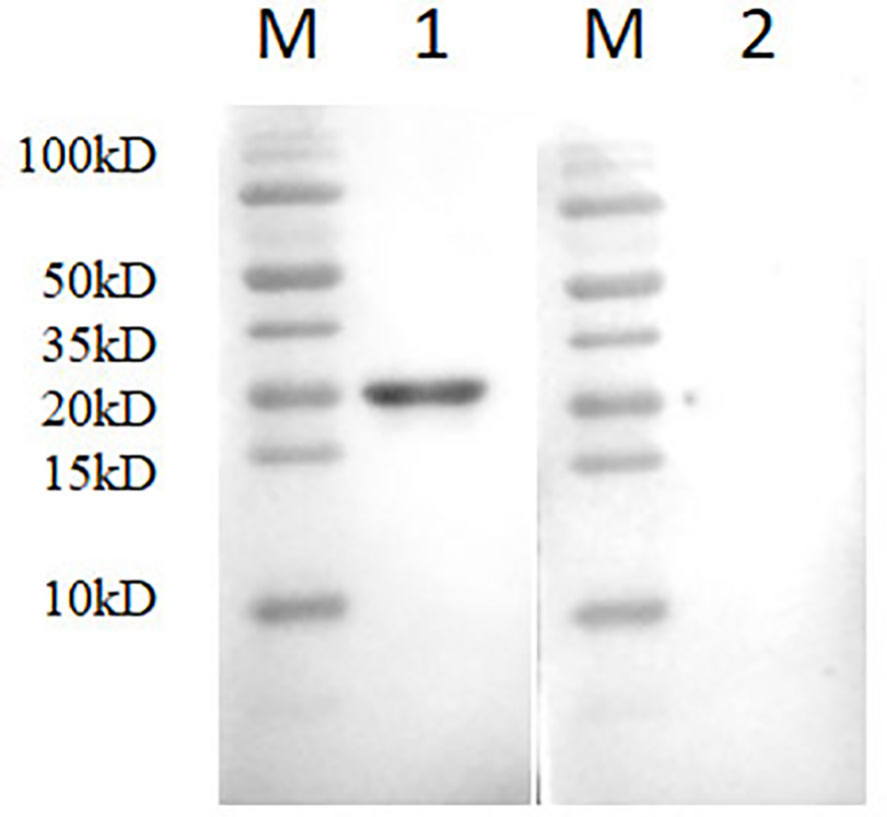
Western blotting analysis using mAb N06. Lane M, protein marker; lane 1, monoclonal antibody N06; lane 2, porcine reproductive and respiratory syndrome virus (PRRSV)-negative serum.

**Figure 3:**
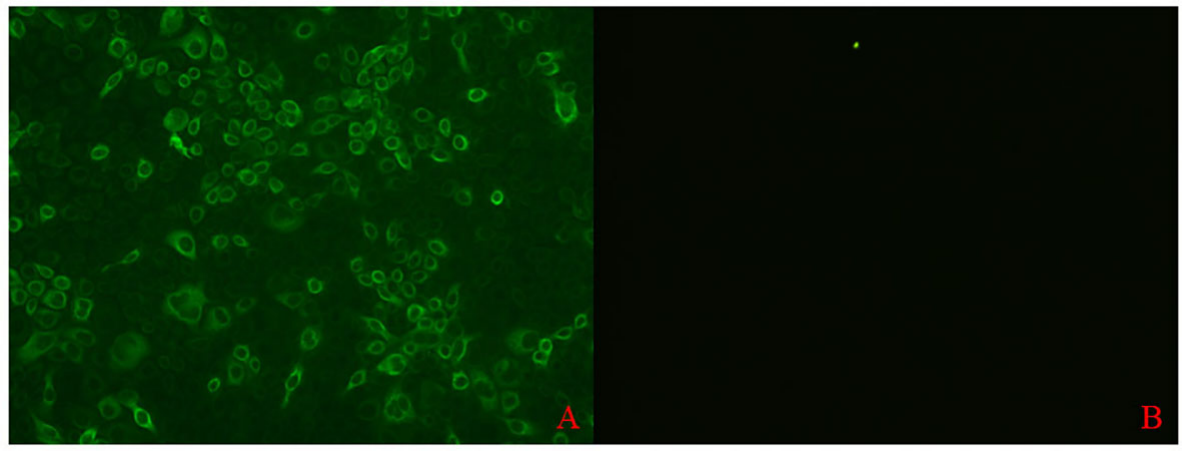
Immunofluorescence assay analysis of monoclonal antibody (mAb) N06 with the native porcine reproductive and respiratory syndrome virus (PRRSV) antigen. **(A)**; mAb N06 reacted with PRRSV-infected cells, **(B)**; PRRSV-negative serum applied as negative control.

A bacterial expression system was used to generate the His-tagged version of the recombinant N protein (**Figure 1A**), which could react with the anti-His-tagged antibody using Western blotting (**Figure 1B**). The purified recombinant N protein was then used to immunize BALB/c mice. The antibodies against the N protein were analyzed using indirect ELISA to ensure that the mice selected for hybridoma production possessed high titers. Cell fusions were carried out and hybridoma cells that secreted anti-N antibodies were selected and subcloned four times by limiting dilution. Finally, a positive mAb clone (N06), which reacted with the recombinant N protein, was selected and cultured on a mass scale to generate ascites fluid through injection into the mice. Western blotting was carried out to evaluate the binding affinity between the mAb and the denatured N protein; the result showed strong reactions (**Figure 2**). The purified ascites fluid reacted with the denatured N protein and showed strong affinity with the PRRSV-infected Marc-145 cells (**Figure 3**). These data suggest that mAb N06 strong affinity the native and denatured forms of the PRRSV N protein.

### Prediction of linear B-cell epitopes

**Figure 4:**
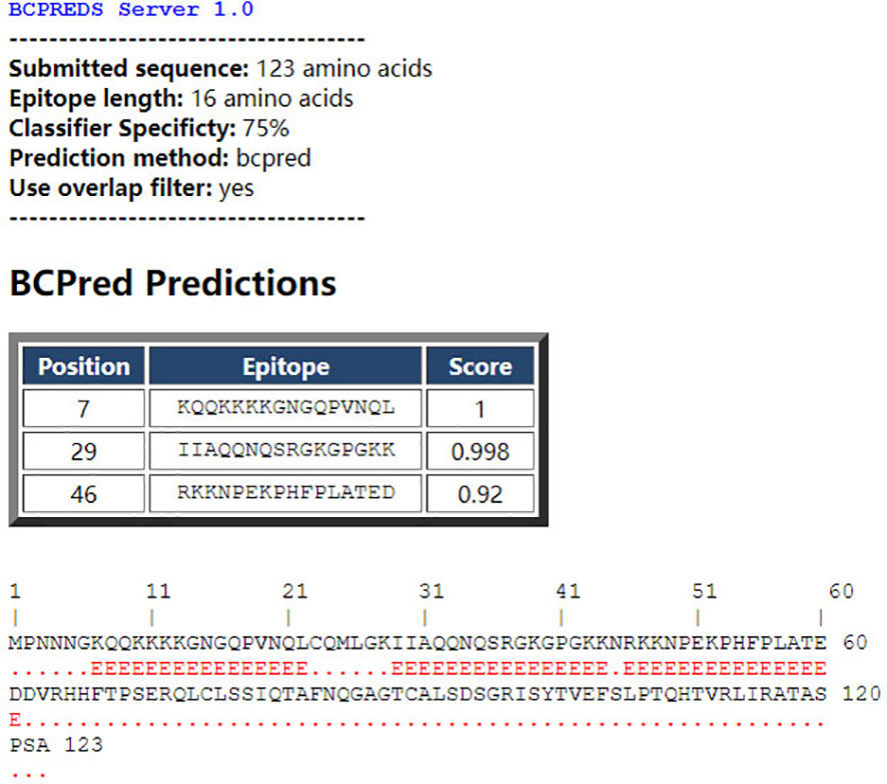
B-cell epitope prediction using the B-cell epitope prediction (BCPred) server. The porcine reproductive and respiratory syndrome virus (PRRSV) N protein amino acid sequence was applied to the BCPred method at a length of 16 amino acids and three peptides. A score of > 0.9 was obtained.

**Figure 5:**
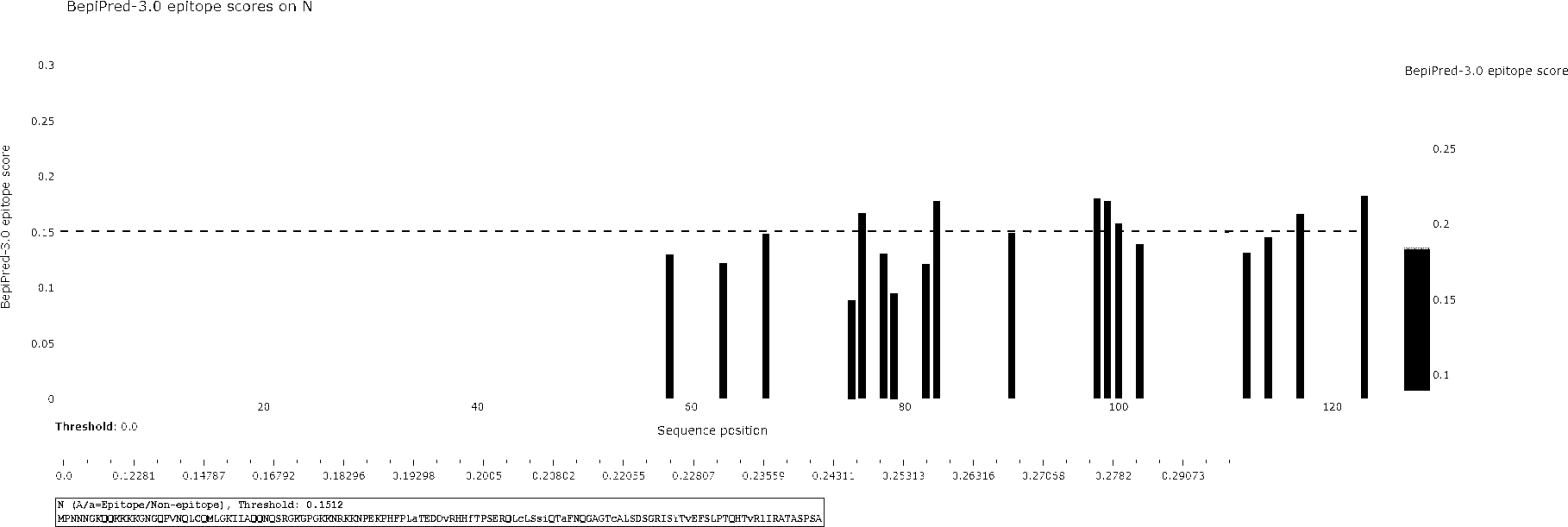
B-cell epitope prediction using the BepiPred-3.0 online software. The porcine reproductive and respiratory syndrome virus (PRRSV) N protein was subjected to BepiPred-3.0 prediction software and the yellow color represents high scores, suggesting the location of the B-cell epitope.

The BCPred software was used to predict antigenic epitopes of the N protein based on accessibility, antigenic propensity, exposed surface, flexibility, hydrophilicity, polarity, and turns. The 123 amino acid protein was analyzed using a frame length of 16 peptides ranging from 12 to 22 amino acids. Fifteen results were obtained and three of them met all the inclusion criteria and had BCPred scores > 0.9, indicating that the N protein most probably consisted of linear epitopes (**Figure 4**). As shown by BepiPred-3.0, two relatively high-score peptides were obtained, and they were mostly distributed in the front part of the sequence, which was similar to BCPred-predicted results (**Figure 5**). To further validate the prediction results, BepiBlast was used and the result showed that two predicted epitopes exist in the N protein, one of which was located from 45 aa to 56 aa, which was close to the previously predicted results.

### Precise identification of B-cell epitope

**Figure 6:**
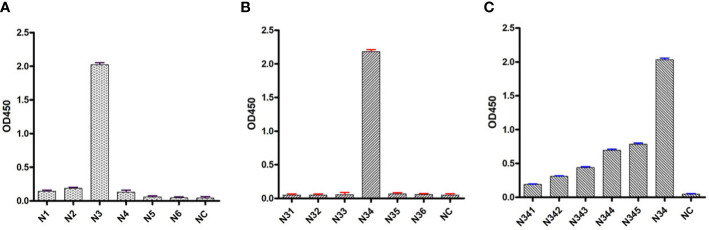
Indirect enzyme-linked immunosorbent assays (ELISA) analysis of synthesized peptides. **(A)**: indirect ELISA for the first series of synthesized peptides. N1–N6 represent truncated peptides covering the porcine reproductive and respiratory syndrome virus (PRRSV) N protein sequence from amino acids 1–25, 20–44, 39–63, 58–82, 77–101, and 96–123, respectively. NC: negative control. **(B)**: indirect ELISA for the second series of synthesized peptides covering the peptide N3. N31: 39–54; N32: 41–56; N33: 43–58; N34:45–60; N35: 47–63. NC: negative control. **(C)**: indirect ELISA for the third series of synthesized peptides covering the peptide N34. N341: 45–55; N342: 46–56; N343: 47–57; N344: 48–58; N345: 49–59; N346: 50–60. NC, negative control.

To determine the B-cell epitope of the N protein, the whole length of the amino acid sequence was first divided into six fragment peptides that overlapped by five amino acids. All the synthesized linear peptides were tested by mAb through indirect ELISA. The results showed that peptide N3 (39–63 aa) possessed the highest absorbance at 450 nm, whereas the other five peptides showed no reactivity with mAb N06, indicating that the potential epitopes were located in peptide N3 (**Figure 6A**). Peptide N3 was then subdivided into five shorter peptides and subjected to indirect ELISA as previously described. According to the result, peptide N34 showed a significantly higher optical density (OD) value than the other four peptides (**Figure 6B**). Subsequently, peptide N34 was further truncated into six 11-aa peptides and screened by indirect ELISA as above. Unlike before, all six peptides showed cross-reactions with mAb N06, with lower OD values than peptide N34, suggesting that peptide N34 might represent all linear B-cell epitopes recognized by mAb N06 (**Figure 6C**).

## Discussion

PRRSV has been one of the most economically damaging pathogens for the swine industry and becomes endemic once introduced into a herd (25). However, the means of pathogenesis, replication, and recognition by the immune system are unclear, and the vaccines that have been developed do not protect pigs against highly pathogenic PRRSV strains (26). Epitope-based vaccines and serological tests require the precise identification of epitopes. Antigens can be represented as both linear and conformational epitopes to the immune system. Linear epitopes are represented by a consecutive series of amino acids in the protein sequence, but only 10% of epitopes are estimated to be linear (27). These linear fragments often constitute portions of conformational epitopes (28).

Previous studies had identified antigenic determinants of PRRSV non-structural proteins, including Nsp2, Nsp10, and Nsp12, yet the highly immunogenic N protein sequence has not been studied in detail (29–31). However, the non-structural proteins are not as important as structural proteins in the virus and their composition ratios are far lower than structural proteins. The antibody levels stimulated by non-structural proteins are too low to be detected. Therefore, they are not ideal diagnostic antigen candidates. Unlike the non-structural protein, the N protein is the major structural protein in PRRSV; the antibodies against the N protein could be easily detected in the early infection stage. In addition, the antibodies against the N protein could remain at a high level for a few months. Current detection methods for PRRSV were developed based on the N protein (32).

In this study, mAbs against the N protein of PRRSV were successfully generated. Since experimental epitope identification is often time consuming and labor-intensive in comparison with computational techniques, prediction software was used for the prediction of linear epitopes of the N protein. Considering that the average length of most antibody recognition sites is 15 amino acids, the parameters of the algorithm were adjusted to search for epitopes ranging from 12 to 22 aa. However, the predicted results using these parameters were equivocal and different software provided different potential linear B-cell epitopes. To identify the complete epitopes, a peptide length of 25 aa was selected for the initial synthesis. The first round of indirect ELISA screening yielded only a single peptide N3 that was recognized by mAb N06, and this sequence was analyzed further. In the following screening ELISA, the truncated peptide N34 retained the activity of its parent peptide N3. All the subdividing N34-generated peptides showed lower activity with mAb N06, indicating that N34 represented a complete linear epitope of the N protein that was recognized by mAb N06. Overall, these experimental results were nearly consistent with the prediction results when the epitope length was 16 aa. This information may be helpful in generating an epitope-based vaccine for PRRSV and also indicates the value of using linear epitope prediction to determine the potential antigenicity of a protein.

## Conclusions

The mAb N06, which reacts directly against the PRRSV N protein, was successfully generated. Synthetic peptides were used to identify a linear stretch of 16 aa, ^45^NRKKNPEKPHFPLATE^60^, that represented a highly immunogenic region of the N protein. This is the first report of the precise identification of the linear B-cell epitope of the N protein that belongs to an isolated PRRSV strain in south China. Owing to the epidemic of PRRSV in local pig farms, this highly specific mAb against the isolated virus strain will be of great help in the development of diagnostic test reagent and effective epitope-based vaccines.

## Data availability statement

The original contributions presented in the study are included in the article/supplementary material. Further inquiries can be directed to the corresponding authors.

## Ethics statement

The animal study was reviewed and approved by Guangdong Laboratory Animals Monitoring Institute Institutional Animal Care and Use Committee (IACUC) (IACUC2021167).

## Author contributions

YC, LX, and MW performed the experiments. MW wrote the draft of the manuscript. FC analyzed the data. LY designed the study. All authors contributed to the article and approved the submitted version.
